# Supplementary material for: Nitrogen fertilization outweighs plant species loss in shaping bacterial belowground diversity in an alpine meadow on the central Tibetan Plateau
Source: FEMS Microbiol Ecol. 2026 Oct 8;102(10):fiag110. doi: 10.1093/femsec/fiag110 (PMC13653335; doi:10.1093/femsec/fiag110)
Supplement: Supplementary file 1 [file fiag110_supplemental_files.zip › Dongmei wu et-al revised-2408-2026 - supplements.docx]

# Supplementary Material

**Nitrogen fertilization outweighs plant species loss in shaping bacterial belowground diversity in an alpine meadow on the central Tibetan Plateau**

Dongmei Wu^a,b^, CiRen Quzong^c^, Zhongjun Jia^d^, Antje Schwalb^b^, Georg Guggenberger^e^**, Shiping Wang^c^, Tsechoe Dorji^c,*^,** Michael Pester ^a,f^

^a^ Leibniz Institute DSMZ—German Collection of Microorganisms and Cell Cultures, Inhoffenstrasse 7B, 38124 Braunschweig, Germany

^b^ Institute of Geosystems and Bioindication, Technische Universität Braunschweig, Langer Kamp 19c, Braunschweig 38106, Germany

^c^ State Key Laboratory of Tibetan Plateau Earth System, Resources and Environment (TPESRE), Institute of Tibetan Plateau Research, Chinese Academy of Sciences, Beijing 100101, P. R. China

^d^ State Key Laboratory of Soil and Sustainable Agriculture, Institute of Soil Science, Chinese Academy of Sciences, Nanjing 210008, P. R. China

^e^ Institute of Earth System Sciences, Section Soil Science, Leibniz Universität Hannover, Herrenhäuser Str. 2, 30419 Hannover, Germany

^f^ Chair of Microbial Physiology, Technical University of Munich, Emil-Ramann-Strasse 4, 85354 Freising, Germany

*Corresponding author: Tsechoe Dorji, E-Mail: tsechoedorji@itpcas.ac.cn, phone: +86 177 1191 5187

## Supplementary Figures

Fig. S1. Experimental plots for this study. The treatments include: no plant removal (Control), removal of *Kobresia pygmaea* (Kp), removal of *Potentilla saundersiana L*. (Ps), and removal of both plants (Kp+Ps). Urea addition and no urea addition.

Fig. S2. Rarefaction curve analysis of amplicon sequencing variants (ASVs) detected by bacterial 16S rRNA gene amplicon sequencing across all analyzed samples.

Fig. S3. Diagnostic plots for the best-selected Linear Mixed Model (LMM) examining the relationship between bacterial alpha diversity and experimental treatments (plant species removal and urea addition). (a) QQ-Plot of residuals: Assesses the normality of residuals. If the points align closely with the reference line, the residuals are approximately normally distributed. (b) Residuals vs. Fitted Values: Evaluates homoscedasticity. A random scatter indicates constant variance, while visible patterns may suggest heteroscedasticity or model misspecification.

Fig. S4. Diagnostic plots for the best-selected Linear Mixed Model (LMM) examining the relationship between soil properties and experimental treatments (plant species removal and urea addition). (a) QQ-Plot of residuals: Assesses the normality of residuals. If the points align closely with the reference line, the residuals are approximately normally distributed. (b) Residuals vs. Fitted Values: Evaluates homoscedasticity. A random scatter indicates constant variance, while visible patterns may suggest heteroscedasticity or model misspecification.

Fig. S5. Diagnostic plots for the best-selected Linear Mixed Model (LMM) examining the relationship between plant species richness and experimental treatments (plant species removal and urea addition). (a) QQ-Plot of residuals: Assesses the normality of residuals. If the points align closely with the reference line, the residuals are approximately normally distributed. (b) Residuals vs. Fitted Values: Evaluates homoscedasticity. A random scatter indicates constant variance, while visible patterns may suggest heteroscedasticity or model misspecification.

Fig. S6. Diagnostic plots for the best-selected Linear Mixed Model (LMM) examining the relationship between soil properties, experimental treatments (plant species removal and urea addition) and bacterial alpha diversity. (a) QQ-Plot of residuals: Assesses the normality of residuals. If the points align closely with the reference line, the residuals are approximately normally distributed. (b) Residuals vs. Fitted Values: Evaluates homoscedasticity. A random scatter indicates constant variance, while visible patterns may suggest heteroscedasticity or model misspecification.

Fig. S7. Diagnostic plots for the best-selected Linear Mixed Model (LMM) examining the relationship between soil properties, experimental treatments (plant species removal and urea addition) and bacterial beta diversity. (a) QQ Plot of Residuals: Assesses the normality of residuals. If the points align closely with the reference line, the residuals are approximately normally distributed. (b) Residuals vs. Fitted Values: Evaluates homoscedasticity. A random scatter indicates constant variance, while visible patterns may suggest heteroscedasticity or model misspecification.

Fig. S8. NMDS ordination of bacterial community composition based on weighted UniFrac distances. Plant species removal treatments include no plant species removal (Control), removal of *Kobresia pygmaea* (Kp), removal of *Potentilla saundersiana* (Ps), and removal of both species (Kp+Ps). Urea treatments are represented as -U (without urea) and +U (with urea).

Fig. S9. Effects of plant species removal and urea addition on the eight most abundant bacterial phyla across the growing season. Plant species removal treatments include no plant species removal (Control), removal of *Kobresia pygmaea* (Kp), removal of *Potentilla saundersiana* (Ps), and removal of both species (Kp+Ps). Urea treatments are represented as -U (without urea) and +U (with urea). Asterisks denote significant effects of dominant plant removal (P), urea addition (U), seasonal variation (V), and their interactions on the relative abundance of the respective phyla (****p* < 0.001, ***p* < 0.01, **p* < 0.05). Only significant results are displayed.
